# Late Neoproterozoic seawater oxygenation by siliceous sponges

**DOI:** 10.1038/s41467-017-00586-5

**Published:** 2017-09-20

**Authors:** Michael Tatzel, Friedhelm von Blanckenburg, Marcus Oelze, Julien Bouchez, Dorothee Hippler

**Affiliations:** 1 Section 3.3: Earth Surface Geochemistry Helmholtz Centre Potsdam GFZ German Research Centre for Geosciences, Telegrafenberg, Potsdam, 14473 Germany; 2 Department of Earth Sciences, Institute of Geological Sciences, Freie Universität Berlin, Malteserstr. 74-100, Berlin, 12249 Germany; 3 Institute of Applied Geosciences Technische Universität Berlin, Ackerstraβe 76, Berlin, 13355 Germany; 4 Present Address: Division 1.1: Inorganic Trace Analysis, BAM, Federal Institute for Materials Research and Testing, Richard-Willstätter-Straße 11, Berlin, 12489 Germany; 5 Present Address: Institut de Physique du Globe de Paris-CNRS, Sorbonne Paris-Cité 1 Rue Jussieu, 75238 Paris 05, France; 6 Present Address: Institute of Applied Geosciences, Technische Universität Graz, Rechbauerstraβe 12, 8010 Graz, Austria

## Abstract

The Cambrian explosion, the rapid appearance of most animal phyla in the geological record, occurred concurrently with bottom seawater oxygenation. Whether this oxygenation event was triggered through enhanced nutrient supply and organic carbon burial forced by increased continental weathering, or by species engaging in ecosystem engineering, remains a fundamental yet unresolved question. Here we provide evidence for several simultaneous developments that took place over the Ediacaran–Cambrian transition: expansion of siliceous sponges, decrease of the dissolved organic carbon pool, enhanced organic carbon burial, increased phosphorus removal and seawater oxygenation. This evidence is based on silicon and carbon stable isotopes, Ge/Si ratios, REE-geochemistry and redox-sensitive elements in a chert-shale succession from the Yangtze Platform, China. According to this reconstruction, sponges have initiated seawater oxygenation by redistributing organic carbon oxidation through filtering suspended organic matter from seawater. The resulting increase in dissolved oxygen levels potentially triggered the diversification of eumetazoans.

## Abstract (web summary)

The Ediacaran–Cambrian oxygenation of seawater is thought to have been caused by lifeforms engaging in ecosystem engineering. Here, the authors show that siliceous sponges increased seawater dissolved oxygen concentrations by redistributing organic carbon oxidation through filtering suspended organic matter.

## Introduction

The Cambrian bioradiation is thought to have occurred when dissolved oxygen concentrations in seawater exceeded a critical threshold of ~5–20 µmol l^−1^, i.e., 2–7 percent of present surface seawater^1^. Rising oxygen levels were thus an important factor for the development of animals on Earth^2^. Ultimately, atmospheric and seawater dissolved oxygen levels increase during the growth of the continental organic carbon reservoir because over > Myr timescales, the CO_2_ flux into the atmosphere–ocean system is balanced by carbon burial which releases oxygen^3^. Suggested triggers for ocean oxygenation during the Ediacaran–Cambrian transition include increased weathering fluxes^4–7^ that would cause an increase in oceanic primary production and organic carbon burial^5, 6^. Enhanced clastic sedimentation during times of high tectonic activity reduces oxidation of organic carbon and causes rising oxygen levels^4^.

Purely biogenic mechanisms might have been responsible for the Ediacaran–Cambrian oxygenation too. A possible terrestrial biogenic trigger of oxygenation is the biological enhancement of phosphorus supply to the ocean by weathering in the Neoproterozoic that resulted in a rise of atmospheric oxygen^8^. A possible terrestrial driver is the development of the fungi-lichen ecosystem that led to the formation of an organic-rich, upper soil layer. This layer restricted the consumption of increased atmospheric oxygen required to weather the subsoil regolith^9^. In the marine realm, a potential trigger of seawater oxygenation was presented in the evolution and expansion of metazoans themselves: some evolutionary innovations would have increased the export of organic matter to the deep sea, and lowered the oxygen demand in shallow water^10^. Suggested mechanisms include an increased efficiency of the biological pump by the evolution of large eukaryotes and rapidly sinking fecal pellets^10^, or the evolution of eumetazoa, enhancing organic carbon transfer to sediment and reducing oxygen consumption in the overlying water column^11–14^. A recent and thus far untested hypothesis states that metazoan development itself caused seawater oxygenation by mechanisms of ecosystem engineering^12^. One proposed mechanism is that benthic filter feeding by sponges has led to seawater oxygenation^12^. By removing large amounts of dissolved and fine particulate organic carbon from seawater, the respiratory oxygen demand was shifted to a greater depth^12, 13^. The ensuing increase in seawater dissolved oxygen concentrations led to enhanced phosphorus burial and P sequestration by sponge symbionts^15^, further decreased primary productivity, and thus led to lower oxygen demands and higher dissolved oxygen levels^12^.

It is challenging to investigate the role of sponges for seawater oxygenation because their impact on the environment depends on their abundance. Unfortunately, because of incomplete sponge spicule preservation, sponge abundance cannot be directly obtained from the Precambrian–Cambrian fossil record. Essentially, all pre-Cenozoic biosiliceous material has been diagenetically altered, with chert (microcrystalline quartz) being the ultimate product of the diagenetic pathway. Cherts are near-ubiquitous in Ediacaran–Cambrian boundary sections, and the presence of sponge spicules suggests that the silicon stems at least partially from the dissolution and reprecipitation of siliceous sponge spicules. Silica diagenesis might even completely obliterate fossil evidence for siliceous sponges, and would have caused the 240-million-year lag time between the origin of silicean spicules (estimated from molecular clock analysis^16^) and their unequivocal identification in the fossil record. Chert occurrence, petrology and geochemistry thus need to be invoked to reconstruct sponge abundance. However, radiolarians^17, 18^, inorganic precipitation of silica from hydrothermal fluids^19^ and seawater^20, 21^ are all potential Si sources for cherts from the Ediacaran–Cambrian boundary, complicating interpretations. The recent demonstration that siliceous sponges often have distinctly different Si stable isotope ratios^22, 23^ implies that siliceous sponge abundance can be reconstructed using Si stable isotopes—provided that the bulk Si stable isotope composition was not altered.

Here we explore one of the most complete stratigraphic sections from the slope-to-basin setting at the SE margin of the Yangtze Platform to determine the abundance of siliceous sponges and to evaluate their biogeochemical impact during the Ediacaran–Cambrian transition. We show that the silicon sources of cherts can be constrained by a multi-proxy approach employing Si stable isotopes, Ge/Si ratios and rare earth elements (REE). The relative abundance of siliceous sponge spicules can be reconstructed using Si stable isotopes. This approach overcomes the challenge of incomplete spicule preservation that has so far impeded testing the hypothesis of seawater oxygenation by (siliceous) sponge proliferation^12^. We determine local changes in the oxidation states of seawater and sediment using Ce-anomalies and enrichment factors (EFs) of redox-sensitive trace metals. We derive variations in the DOC pool size from Ge/Si of clays and Y/Ho ratios. Our data suggests that siliceous sponges with low oxygen demands^24^ expanded on the continental slope across the Ediacaran–Cambrian boundary. These expanding sponge communities shifted the respiratory oxygen demand from the water column to the water-sediment interface by organic matter filter feeding. The resulting shift in oxygen demand to greater water depth led to increased P burial, resulting in lowered primary productivity and thus lowered oxygen consumption.

## Results

### Geological setting and samples

**Fig. 1:**
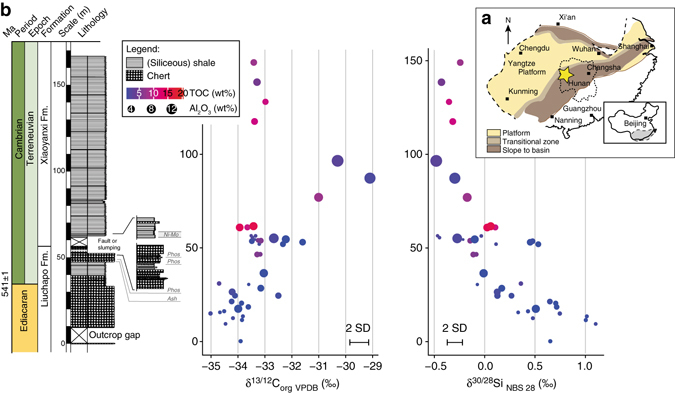
Lithostratigraphic profile of the Lijiatuo section and C- and Si stable isotope ratios of chert and siliceous shales and the paleogeographic setting of the site. The Lijiatuo section **a** is located in the continental slope-to-basin setting at the Yangtze Platform **b** and contains chert, siliceous shales and regional, Lower Cambrian marker horizons, i.e., phosphate bearing units (Phos) and a Ni-Mo layer as well as an ash layer (Ash) dated to 546 ± 12 Ma^26^. The sedimentary facies of the section changes towards higher TOC concentrations as shown by symbol colour (*blue*: <5 wt%, *red*: 15 wt%) as well as towards higher the clay-mineral contents (represented by the Al_2_O_3_ concentration, increasing symbol size). *Scale bar*, 10 m

The Lijiatuo section is an almost completely exposed section on the Yangtze Platform, Hunan Province (Fig. 1a) that straddles the Ediacaran–Cambrian boundary. Recent evidence from litho- and chemostratigraphy^25^, as well as U-Pb age constraints^26^ allow for the integration of the Lijiatuo section into the Ediacaran–Cambrian stratigraphic framework of the Yangtze Platform^25^, placing its depositional history between ~550 and 525 Ma. The Lijiatuo section comprises the Liuchapo Fm. and the lower part of the Xiaoyanxi Fm., both representing deep-water depositional environments below storm wave base at the continental slope to basin. The sedimentary facies change from mostly pure chert to organic-matter-rich siliceous shales (Fig. 1b). The bottom of the section is dominated by black, bedded cherts with total organic carbon (TOC) concentrations <1 wt%, where the upper part is characterized by mostly phosphatic-siliceous shales and finely laminated organic-rich black shales with TOC concentrations of up to 15.1 wt% (Supplementary Data 1). The predominant minerals are quartz and illite. Some samples contain  > 5 wt% barite. Pyrite, K-feldspar, Ba-feldspar and an unspecified 15 Å clay mineral occur locally as accessory phases (Supplementary Data 2). The lithostratigraphic and formational boundary is composed of phosphate-nodule bearing siliceous rocks and organic-rich shales. These shales are partly enriched in redox-sensitive elements (Supplementary Data 3), such as in the Ni and Mo-rich layer^27^ that is regionally exposed in the lowermost black shales of the Xiaoyanxi Fm. on the Yangtze Platform. Despite an overall low fossil content, fossil sponges or sponge spicules are present in both formations^28^, with abundant sponge spicules in some layers, particularly in the Xiaoyanxi Fm. Some samples from the Liuchapo Fm. contain spherical objects, i.e., putative radiolarians.

### Geochemical data

**Fig. 2:**
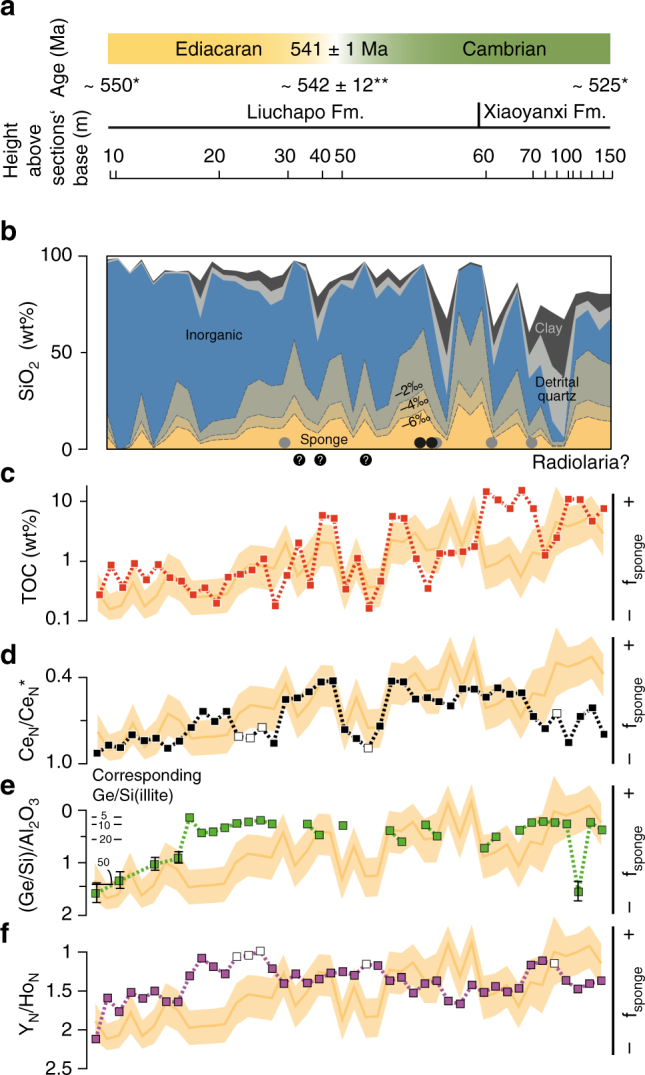
The estimated siliceous sponge spicule abundance and geochemical records across the Ediacaran–Cambrian boundary at the Lijiatuo section. **a** Stratigraphic context and age of the Lijiatuo section based on stratigraphic records from South China^25^(*) and a dated ash layer^26^(**); **b** Abundance of Si-bearing components calculated by a geochemical mass balance. Colour shading at the bottom indicates several estimates for the abundance of sponge-derived- and inorganic silica based on assumptions of ε^30^Si_(sponge-seawater)_ between −6‰ and −2‰. Occurrences of sponge spicules and putative radiolarians, i.e., round objects (Supplementary Fig. 5) are indicated by black circles (our samples) and gray circles^28^. **c** TOC concentrations; **d** Ce_N_/Ce_N_* (Ce-anomalies); open symbols mark samples with Pr_N_/Yb_N_ > 1, indicating flat, non-seawater REE patterns; **e** Al_2_O_3_-normalized Ge/Si and corresponding Ge/Si of illite; **f** Y_N_/Ho_N_ ratios. For better visibility, samples are plotted with an equidistant X axis. In **c**-**f** f_sponge_ (*yellow shades*) shows the abundance of Si in sponges relative to total non-detrital Si-bearing components that is independent of ε^30^Si_(sponge-seawater)_. Uncertainty envelopes of f_sponge_ are calculated from uncertainties in endmember estimates and a Monte Carlo procedure, and represent 50% confidence intervals (Supplementary Data 6)

The silicon stable isotope ratio decreases from 1.1‰ *δ* ^30^Si at the base of the section to −0.5‰ *δ* ^30^Si at its top (Supplementary Data 4). The sedimentary sponge spicule abundance, calculated from δ^30^Si values (see Methods) (Fig. 2b) shows an overall increasing trend across the Lijiatuo section. Likewise, the relative sponge spicule abundance, f_sponge_, defined as the sponge-derived Si relative to the sum of Si derived from non-detrital components (cf. Methods) (yellow shade in Fig. 2c–f) shows an overall increasing trend across the Lijiatuo section. TOC concentrations vary along with f_sponge_ (Fig. 2c). Ce_N_/Ce*_N_ is ~1 at the base of the section and drops to values as low as 0.42 and overall follows variations in f_sponge_ (Fig. 2d). Ge/Si_(illite)_ (see Methods) decrease in the lowermost 20 m of the section from  > 50 to <5 and remain relatively constant above. Similarly, Y_N_/Ho_N_ ratios initially decrease from 2.1 to 1.0 and mostly remain below 1.5 (Fig. 2f).

### The primary silicon isotope signature of chert

The silicon isotope record of cherts and siliceous shales can provide information about silicon sources provided that the isotopic signature has not been significantly modified during early diagenesis of sedimentary opal (i.e., dissolution of some fraction of the biogenic Si) and late diagenesis when silica phase transformations occur.

Chert precursors, such as siliceous sediments can shift in their bulk silicon isotope ratios during early diagenesis by selective dissolution in silicon-poor water^29^. However, this process was subdued in the Precambrian when seawater silicon concentrations were close to the opal-A saturation at ~28 p.p.m. Si^30^. Sponges and radiolarians were the first organisms of sufficient abundance to utilize silicon. Upon their first appearance seawater silicon concentrations were still high^31^, and are thought to have remained close to these levels, as suggested by the absence of an increase in bulk chert δ^30^Si over ~ 25 Myrs (see discussion below). Thus, dissolved silicon concentration remained high until the early to middle Paleozoic radiation of sponges and radiolarians^32^. Dissolved silicon high in concentration may even have diffused into sediments and led to inorganic opal-CT precipitation^30^, or to microbially mediated opal-A precipitation^33^. The high silicon concentration in seawater thus hampered early diagenetic Si loss from Precambrian and Paleozoic marine sediments and ensured that the primary Si stable isotope signature of the deposited siliceous sediments was not shifted during early diagenesis.

Exchange of silicon during diagenetic phase transformation (from opal-A to opal-CT to quartz) can potentially affect the silicon isotope composition of chert. Geilert et al.^34^ recently demonstrated that opal-CT in fossil volcanic sinter deposits was systematically 1.3‰ lower in δ^30^Si than opal-A sinter deposits from the same location. This observation suggests substantial ^30^Si-loss during opal-A to opal-CT transformation. An alternative and still untested explanation for this observation, however, is that the initial δ^30^Si value differed between these two phases. We suggest that the high-SiO_2_ siliceous sediments studied here have been resistant to Si stable isotope shifts during diagenesis. One reason is a simple mass balance consideration: to shift the bulk chert δ^30^Si requires unrealistically high mass losses or Si exchange, given that Si is the major constituent of the rock. Another is that during silica phase transformations, the exchange of Si between sedimentary silica layers with high and variable clay mineral contents is predicted to be negligible. Firstly, exchange of fluids between sediment layers is not favoured by the presence of impervious clay-rich layers. Secondly, the kinetics and hence burial depth of the silica phase transformations depend on detrital mineral concentrations^35^. This means that a stack of siliceous sediment with variable detrital mineral contents transforms to higher silica polymorphs (opal-CT and quartz) with different rates during burial. The ensuing differences in the timing of silica phase transformations impede the exchange of Si between sediment layers. These conditions act in favor of preserving the primary bulk silicon isotope composition of siliceous sediments deposited in siliciclastic depositional environments. This inference is supported by the preservation of isotopic heterogeneity over centimeter- to decimeter scales in our samples. Dissolution-reprecipitation will, however, affect silicon isotope ratios at the microscale^36^. We conclude that any diagenetic shift in the silicon isotope composition of our samples is likely negligible and that bulk rock silicon isotope ratios can be interpreted in terms of paleo-environmental conditions.

### Seawater and siliceous sponge spicules as silicon source

Silicon isotopes, REE patterns, Ge/Si ratios, and petrographic evidence suggest that inorganic precipitates from seawater and siliceous sponge spicules are the dominant Si constituents. In contrast, the fraction of Si hosted by clay minerals and detrital quartz is variable and lower (mostly < 50%).

High δ^30^Si values at the sections’ base (Fig. 1b) suggest inorganic silica precipitation from seawater, in line with previously reported δ^30^Si values from Ediacaran inorganic cherts^21^. The oceans’ dissolved silicon is dominantly supplied by rivers^37^ that carry silicon depleted in ^28^Si by clay formation during continental weathering^38^. Modern rivers thus carry dissolved Si with an average δ^30^Si value of 1.28‰^39^. Moving upsection, the trend towards lower δ^30^Si suggests a progressive contribution of silica from siliceous sponges (and possibly radiolarians, as discussed below) that contain silicon with a low δ^30^Si signature^22, 23^. The Si stable isotope fractionation in modern sponges is thought to be controlled by Si influxes into and effluxes out of sclerocyte cells where Si isotopic fractionation, denoted by ε^30^Si (ε = α−1) attains a constant value at high seawater dissolved Si concentrations^22, 23^. We thus assume a constant ε^30^Si_(sponge-seawater)_ for Precambrian sponges, because sclerocyte cells existed already before 580 Ma^40^ and seawater had a high Si concentration^30, 32^.

We can discount an increasing contribution of ^28^Si-rich hydrothermal silicon upsection based on REE patterns and Ge/Si. REEs, scavenged from seawater into authigenic phases, consistently show seawater-like patterns with Eu/Eu* ~ 1, variably negative Ce anomalies and Y_N_/Ho_N_ > 1 (Supplementary Fig. 1 and Supplementary Data 5). The Ge/Si ratios of pure chert samples range between 0.4 and 0.8 µmol mol^−1^ (Supplementary Fig. 2 and Supplementary Data 3), similar to Precambrian seawater silica precipitates at 0.25–0.8 µmol mol^−1^ ^21^ and modern seawater at 0.7 µmol mol^−1^, but much lower than hydrothermal fluids at ~7 µmol mol^−1^ ^41^.

We can also discount that the trend in decreasing δ^30^Si (cf. Supplementary Data 4) is caused by clay mineral or possible TOC abundance-controlled variations in the Si stable isotope fractionation factor for silica precipitation (Supplementary Note 1). This independence is supported by the constant δ^30^Si values for multiple bulk chert samples from within two stratigraphic layers with variable TOC and Al_2_O_3_ concentrations (Supplementary Fig. 3). We note that ^28^Si could potentially be enriched in sediment through preferential ^28^Si adsorption onto Fe-Mn oxyhydroxides. Reactive Fe-Mn surfaces might be responsible for the sedimentary trace metal enrichment (see discussion below). This effect however cannot explain the δ^30^Si decreases in the lowermost part of the section where trace elements are not enriched. No correlation between trace element enrichment and δ^30^Si can be observed (see Supplementary Fig. 4).

An underlying assumption of our Si isotope mass balance is that the oceanic Si inventory was sufficiently large^30^, such that neither its Si concentration nor its δ^30^Si changed due to biogenic silica precipitation. This assumption is supported by the continuous decrease in δ^30^Si values over a period of ~25 Myrs along the entire section. If biogenic silica precipitation were significant relative to that of inorganic silica δ^30^Si would be expected to transiently increase. If the output flux of silica through siliceous sponges had been constant and high, in contrast, seawater would attain a lower silicon concentration and a higher steady state δ^30^Si value. As a consequence, the δ^30^Si of sponge spicules would increase within a few million years and bulk chert δ^30^Si would increase along the section. We conclude that only the abundance of siliceous sponge spicules and potentially some minor radiolarians are the cause of the low δ^30^Si Si in chert and siliceous shales, as supported by petrographic evidence for sponge spicules and putative radiolarians (Fig. 2b and Supplementary Fig. 5). Therefore, the shift in δ^30^Si allows for the determination of sedimentary sponge spicule abundance by mass balance (see Methods).

We observe that the relative abundance of sponge spicules, f_sponge_, defined as sponge-derived Si relative to the sum of Si derived from non-detrital components (cf. Methods), steadily increases throughout the Ediacaran–Cambrian Liuchapo Fm. and remains high in the early Cambrian Xiaoyanxi Fm. (Fig. 2), suggesting siliceous sponge proliferation along the continental slope across the Ediacaran–Cambrian boundary. It should be noted that while the estimated amount of Si derived from sponges depends on both the prescribed seawater δ^30^Si value and on the sponge Si stable isotope fractionation (Fig. 2b), temporal trends in f_sponge_ are unaffected by these estimates, provided they remained constant (Fig. 2c–f). Details of the mass balance are reported in Supplementary Note 2, and the estimated uncertainty on f_sponge_ in Supplementary Data 6).

### Diagenetic preservation of TOC and δ^13^C_org_

TOC concentrations increase upsection from ~0.2 to 15.1 wt%, while δ^13^C_org_ increases from −35 to −29‰ up to 100 m and decreases higher up in the section to −33.1 to −33.5 δ^13^C_org_ (Fig. 1b). From the covariation of organic carbon concentrations with f_sponge_ (Fig. 2c), we suggest that organic carbon transfer rates increased at the same time as siliceous sponges became more abundant. A more efficient biological pump would have led to higher organic carbon burial fluxes. The resulting anoxia would then have enhanced the organic carbon burial efficiency. The simultaneous increase of δ^13^C_org_ (Fig. 1a) supports enhanced organic carbon burial efficiency, as burial of isotopically light organic carbon increases the residual seawaters’ δ^13^C. The higher organic carbon burial efficiency led to increased δ^13^C_org_, simultaneously across a range of slope-to-basin settings on the Yangtze Platform^42^. This observation is compatible with increased atmospheric oxygen concentrations that oxygenated seawater at short time scales < 1 kyr.

**Fig. 3:**
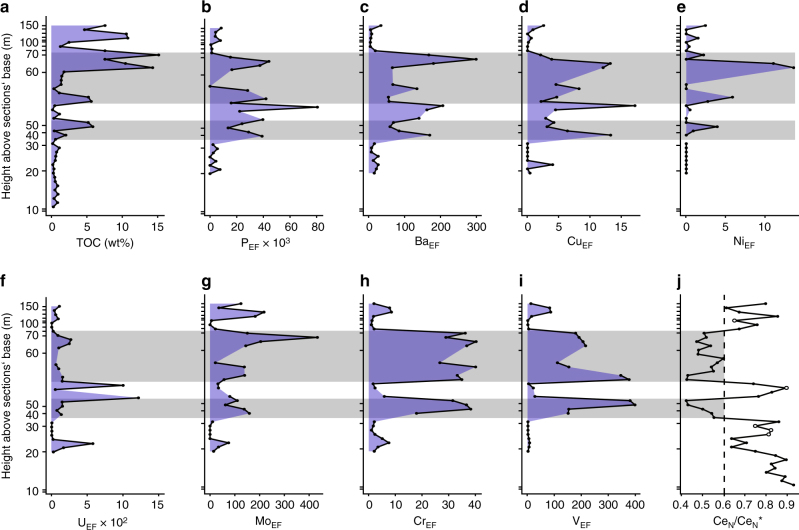
TOC concentrations, EFs (Al and PAAS-normalized element concentrations) of redox-sensitive trace elements and Ce_N_/Ce_N_* at the Lijiatuo section. **a** Total organic carbon concentrations; **b**–**i** EFs of P, Ba, Cu, Ni, U, Mo, Cr and V are highest in organic-rich sediments and suboxic water as indicated by Ce_N_/Ce_N_* (**j**, horizontal *gray shades* indicate the most oxic conditions, i.e., Ce_N_/Ce_N_* < 0.6). *Open symbols* mark samples with Pr_N_/Yb_N_ **> **1, indicating flat, non-seawater REE patterns (cf. Fig. 1). Samples with <3% detritus are not shown due to potential normalization artifacts resulting from propogation of analytical uncertainties^43^

Our proxy data provide evidence that TOC and δ^13^C_org_ were not diagenetically modified. If significant post-depositional organic carbon oxidation had occurred, TOC concentrations would be underestimated and δ^13^C_org_ shifted (note that in this analysis we excluded samples that have low TOC concentrations in which even minor loss of isotopically fractionated C can be manifested in bulk rock C isotope ratios; see Supplementary Note 3 and Supplementary Fig. 6). However, the significant correlations between TOC and both the Ni and the Cu EFs, respectively (Fig. 3), suggest limited post-depositional organic carbon oxidation, as organic matter is less resistant to bacterial remineralization compared to Ni and Cu that are co-deposited at high organic matter fluxes and immobilized when reducing conditions are met^43^. If post-depositional organic carbon losses were indeed low, δ^13^C has not been substantially altered either.

### Seawater oxygenation during sponge expansion

The TOC and δ^13^C evidence for enhanced organic carbon burial in deep water depositional settings is corroborated by evidence for the corresponding decrease in the DOC pool size, and for the redox states in seawater and sediment.

Ge/Si_(clay)_ is diagnostic of the DOC concentration in seawater^20, 44^. During initial sponge expansion, the Ge/Si ratios of clay minerals (estimated assuming Ge/Si of silica = 0.5 and using Al_2_O_3_ concentrations; Supplementary Fig. 2) continuously decreased (from ~50 to 10 µmol mol^−1^; Fig. 2e). This observation suggests that the DOC pool size decreased. The rationale for this interpretation is that Ge forms organic complexes through chelation with organic ligands^45^ that subsequently adsorb onto clay mineral surfaces. Therefore, clays obtain increasing Ge/Si with increasing DOC concentration in seawater. Bulk sediment Ge/Si ranges from 0.43 to 5.04 µmol mol^−1^ and increases with clay mineral abundances (Supplementary Fig. 2). The calculated Ge/Si ratios of illite, the predominant clay mineral in our Late Ediacaran samples (Supplementary Data 2, 7), are in the range of Lower Ediacaran (Doushantuo Fm.) chert nodules (~20–400 µmol mol^−1^), indicating DOC-rich seawater^44^. A decrease in Ge/Si during the latest Ediacaran to Lower Cambrian to Ge/Si < 20 µmol mol^−1^, similar to modern clay values (up to 24 µmol Ge per mol Si^46^) and similar to Ediacaran–Cambrian cherts (between 1 and 10 µmol Ge per mol Si^20^) suggests a decreasing DOC pool size.

A decreasing DOC pool size during the Precambrian–Cambrian transition is also supported by Y_N_/Ho_N_ ratios that decrease from ~2.1 to 1.0 (Fig. 2f). Fractionation of Y from Ho in seawater is controlled by surface complexation onto inorganic metal oxide functional groups or organic substances^47^. The ligands for surface complexation are predominantly provided by organic particles^48^ and for most organic ligands Ho is preferentially complexed over Y^49^.

Contemporary seawater oxygenation is suggested by negative Ce anomalies (Ce_N_/Ce*_N_ < 1), i.e., a redox-controlled Ce-deficiency relative to Pr and Nd in bulk rock (Fig. 3c). Ce anomalies show decreasing values with increasing f_sponge_ (Fig. 2d). The primary source of REEs, including Ce, is seawater from which REEs are scavenged by authigenic mineral formation at the sediment–water interface^50^ (Supplementary Note 4). Ce_N_/Ce*_N_ records the water redox state, as indicated by typical seawater REE patterns in bulk sediment (Supplementary Fig. 1). The Ce_N_/Ce*_N_ ratio is ~1 at the sections’ base and decreases to values as low as 0.42 at the top of the Liuchapo Fm. Ce_N_/Ce*_N_ in seawater decreases when soluble Ce(III) is oxidized to insoluble Ce(IV) relative to the adjacent REEs^51^. Decreasing Ce_N_/Ce*_N_ over the terminal Ediacaran documented here for the slope-to-basin setting at the Lijiatuo section has also been recorded in contemporaneous shallow-water carbonates of the Dengying Formation^52^. This trend suggests that this seawater oxygenation trend has at least a regional, if not a spatially even larger significance.

High EFs relative to the Post-Archean Australian Shale (PAAS)^53^ of redox-sensitive elements indicate reducing conditions. EFs are as high as 17 (Cu), 14 (Ni), 1218 (U), 433 (Mo), 40 (Cr) and 397 (V) (Fig. 3). Notably though, the highest EFs and thus most reducing sedimentary conditions are observed in samples with a substantial Ce-anomaly, i.e., where conditions in the seawater immediately above the sediment were oxidizing. This apparent conflict between redox-proxies is well known in Ediacaran to lower Cambrian cherts. It was suggested that a biogenic silica source derived from the oxic part of the water column was the carrier of low Ce_N_/Ce*_N_ to reducing sediments^17^. However, silica is not a major carrier of REE (Supplementary Fig. 7). Another possibility is that enhanced transfer of organic carbon from suboxic seawater to sediment reduced dissolved oxygen consumption in seawater. The higher oxygen levels led to oxidation of Fe and Mn and formation of Fe-Mn oxyhydroxides in the water column. V and Cr (and also other trace metals) were then attached to Fe-Mn oxyhydroxide surfaces and transferred to sediment. At the same time high TOC caused reducing conditions in the sediment where V and Cr, unlike Fe and Mn, were immobilized by complexation with humic and fulvic acids^43^. The V_EF_- Ce_N_/Ce*_N_ and the Cr_EF_- Ce_N_/Ce*_N_ relationships (Fig. 3h, i) substantiate the link between organic matter burial, oxygen increase and coupling to the redox cycling of Mn and Fe^43^. High organic matter fluxes and enhanced Fe-Mn-oxyhydroxide shuttling of redox-sensitive metals in suboxic water to reducing sediments thus provides an explanation of the simultaneous enrichment of trace metals in sediments (V_EF_ and Cr_EF_) and oxygenation of bottom water (Ce_N_/Ce*_N_), highlighting the redox contrast between seawater and sediment.

Such redox contrast between sediment and the overlying water column would lead to a redox gradient within shallow sediment. We argue that the high EFs of P (up to 80,000) and Ba (up to 300) (Fig. 3b, c) reflect their retention at the suboxic water–sediment interface. The simultaneous enrichment of Ba and P can be caused by both high productivity and by increasing oxygen levels in sediment^54^. Ba immobilization as barite occurs at the sulfate/sulfide redox interface^55^ and P accumulates under (sub-)oxic conditions through the bacterial formation of refractory P-compounds^56^. We argue that at the Lijiatuo section Ba- and P enrichment is primarily due to an oxygen increase in shallow sediment in contact with suboxic seawater. The reason is that Ba is present in form of diagenetic barite fronts that indicate Ba mobilization in underlying, reducing sediments, upward migration and precipitation at the sulfate/sulfide redox interface in shallow sediment. The correlation of P- and Ba enrichment thus suggests that P-immobilization is redox-controlled as well and occurred close to the water-sediment interface. The cumulative evidence for enhanced carbon burial efficiency, a decreased DOC pool, and higher dissolved oxygen concentrations during increased sponge spicule deposition suggests a causal relationship.

### Filter feeding sponges as trigger for seawater oxygenation

A growing population of siliceous sponges on the Late Precambrian continental slope provides an explanation for the redox changes deduced from our geochemical proxy data. According to the hypothesis by Lenton et al.^12^ sponges have reduced the respiratory oxygen consumption in shallow seawater by removing DOC and fine POC (<10 μm^57^) from seawater by filter-feeding^58^. As a result, they shifted the oxygen demand by organic carbon oxidation to greater depth. This mechanism resulted in a net transfer of organic carbon to sediment and had large effects on seawater geochemistry^13^. Our data suggest that these effects included enhanced transfer of organic carbon from seawater to sediment, redox-sensitive trace metal enrichment in sediment, a decrease in the DOC pool size and increased dissolved oxygen levels. Seawater oxygenation might have been amplified by redox-controlled P immobilization and possibly by P-sequestration by sponge symbionts^15^, resulting in reduced dissolved P concentrations in seawater that limited primary productivity and oxygen demand by oxidation of organic carbon even further^12^.

### The causality dilemma of oxygen and sponges

Increased oxygen levels would thus have been triggered by increased organic carbon extraction from seawater through filter feeding by sponges. However, it is unclear whether sponges expanded in oxygen-poor seawater and caused an increase in oxygen levels, or whether sponges rather expanded as consequence of seawater oxygen concentrations exceeding their minimum requirements.

If sponge expansion was impeded by a lack of oxygen in seawater, an initial increase in oxygen levels must have occurred, triggering their expansion. One possible trigger is a sea level fall in an ocean stratified with respect to dissolved O_2_ that would shift the redoxcline relative to the depositional site. This shift would cause a change in the sedimentary facies and in the organic carbon isotope composition. δ^13^C_org_ would be up to 2‰ lower through the decreasing contribution of organic carbon derived from chemoautotrophs^59^. We consider a falling sea level as cause for oxygen increase at Lijiatuo section unlikely, because positive shifts in δ^13^C_org_ have been observed in different paleo water depths on the Yangtze Platform^42^. Moreover, a regression-related positive δ^13^C_org_ shift conflicts with globally increasing δ^13^C records^60^ and a simultaneously rising global sea level^61^. On the Yangtze platform, this rising sea level is documented in transgressive system tracts in shallow water sections^62^.

We rather suggest that the observed facies change and increase in δ^13^C_org_ result from sponge expansion. Sponge expansion would increase the organic carbon burial efficiency, deplete seawater ^12^C, and raise the δ^13^C of subsequently produced organic matter. Some modern sponges can tolerate oxygen concentrations as low as 0.5–4% of present atmospheric levels, i.e., <11 µmol l^−1^ O_2_ for at least a portion of their life cycle^24^, giving further credibility to the potential role of sponges as triggers the Late Proterozoic oxygenation^12, 63^.

### Potential effects of radiolarians and non-siliceous sponges

The silicon isotope record at Lijiatuo section is also compatible with deposition of radiolarians that carry ^28^Si-enriched silicon in their tests. Upon their death, radiolarians would have led to the rapid transfer of organic carbon to sediment. Therefore, radiolarians and sponges potentially affect the δ^30^Si and redox records alike and might have both contributed to seawater oxygenation. While the Si stable isotope fractionation by radiolarians is poorly constrained, it is known that modern radiolarians do not fractionate ^28^Si to the same extent as sponges^64^. A fractionation factor that is lower than that for sponge spicule formation implies that radiolarian tests are present in higher absolute abundance in chert (cf. Fig. 2b). This implication is not supported by the petrographic evidence. Radiolarian tests and sponge spicules might have both been deposited. We note, however, that the petrographic evidence for sponge spicules together with the good TOC- f_sponge_ correlation (Fig. 2c) suggests a high fidelity of f_sponge_ as a proxy for the siliceous sponge spicule abundance. While radiolarians might have contributed to enhanced organic carbon burial and seawater oxygenation, their presence is not unequivocally shown by fossil evidence (Supplementary Fig. 5), and therefore we suggest that sponges were the decisive ecosystem engineers.

We note that the proliferation of non-siliceous sponges, undetectable by Si stable isotopes, might have contributed to the observed changes in redox conditions too. Again, the overall good TOC- f_sponge_ correlation suggests that sponges with siliceous spicules were the predominant drivers of enhanced carbon transfer to the benthos. Their predominance is likely in light of high Si levels in Precambrian seawater^65^, as even in modern, Si-depleted seawater ~75% of sponges build siliceous spicules^57^.

### The changing biogeochemistry of marine ecosystems

**Fig. 4:**
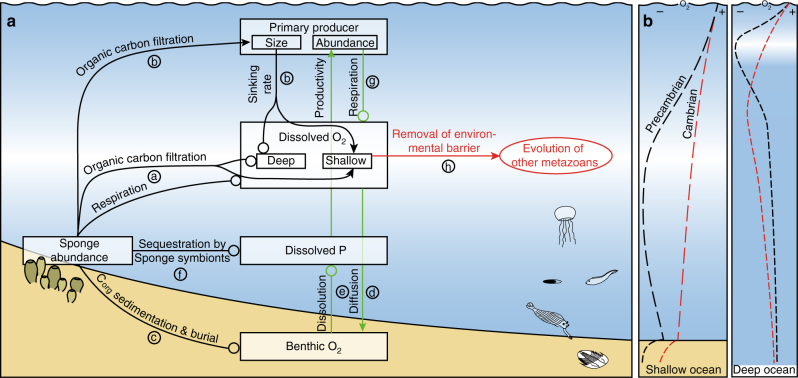
Couplings in the Late Neoproterozoic to Cambrian marine ecosystem and their effects on the oxygen concentration in the water column. **a** Effects resulting from the expansion of sponges. Processes a–h are denoted by *arrows* and are detailed in the text. *Arrows* denote positive couplings (enhancing mechanisms) and *circles* negative couplings (inhibiting mechanisms). **b** With expansion of sponges the oxygen minimum zone shifted towards greater water depth, increasing the benthic oxygen inventory at the continental slope

With the appearance of filter-feeding benthic sponges, the biogeochemistry of the Late Neoproterozoic marine ecosystem changed fundamentally^13^. In Fig. 4, we summarize processes (a–h) that according to our model emerged as a result of sponge expansion and ensuing seawater oxygenation in the Late Precambrian.

Filtration of organic matter from seawater resulted in increased dissolved oxygen levels in shallow water (process a) through the shift in oxygen demand to depth (Fig. 4b). The selective removal of DOC and fine POC promoted the evolution of larger, eukaryotic primary producers, increasing organic carbon sinking rates and lowering re-oxidation rates in the upper water column^12^ (process b), contributing to the descend of the oxygen minimum zone (Fig. 4b). Enhanced organic carbon transfer to depth (process c) led to reducing conditions in sediment and subsequently to enhanced carbon burial that might have increased atmospheric oxygen concentrations. Diffusion of oxygen-rich seawater into sediments (process d) caused the establishment of a redox gradient close to the water-sediment interface. Under (sub-) oxic conditions, i.e., non-zero oxygen concentrations at the water-sediment interface, phosphorus was immobilized through the bacterial formation of refractory P-compounds^56^, (process e). Moreover, P-sequestration by sponge symbionts^15^ would have contributed to reduced levels of dissolved P (process f). As P is the limiting nutrient on geological time scales^66^, the reduced dissolved P inventory limited primary productivity, the sinking flux of organic matter and thus decreased the respiratory oxygen demand in seawater^12^ (process g). These couplings (between benthic oxygen concentration, dissolved P concentration, primary productivity and dissolved oxygen levels) created a positive feedback loop (*green arrows*) that led to a more oxygenated ocean. While full ocean-atmosphere oxygenation has extended until the emergence of land plants^67^, the seawater dissolved oxygen increase related to siliceous sponge expansion has potentially exceeded a necessary threshold for the evolution and distribution of animals with high oxygen demands^63^ (process h) and might thus have been critical for the Cambrian bioradiation. With the subsequent invention of bioturbation oxygen levels declined in Cambrian Stages 3 and 4 (~ 521 to ~ 509 Ma)^68^.

We have suggested that the appearance of sponges has ultimately raised seawater dissolved oxygen levels by reducing sedimentary P-recycling and primary productivity. Whether these processes also led to atmospheric oxygen increase depends on whether the O_2_ availability from enhanced organic carbon burial overcompensated the reduction in O_2_ production that resulted from reduced primary productivity caused by lowered P-levels in seawater.

## Methods

### Silicon stable isotope analysis

Silicon stable isotope ratios were determined on a Thermo Neptune multi-collector inductively coupled plasma mass spectrometer (MC-ICP-MS) equipped with a Neptune Plus Jet Interface at GFZ Potsdam following protocols detailed in Oelze et al.^69^. Samples were diluted to 0.4–1 p.p.m. Si, doped with matching concentrations of Mg and introduced into an ESI APEX desolvator. Analyses were made in medium- or high resolution mode with typical signal intensities > 10 V on a Faraday cup (10^11^ Ω) for ^28^Si; blank intensities were usually < 5 mV. Instrumental mass bias correction was achieved by standard-sample bracketing after internal correction of mass bias drift by Mg isotope ratios. The ^29^Si/^28^Si and ^30^Si/^28^Si isotope ratios are reported as per mil deviation from the international reference material NIST 8546 *aka*. NBS 28, i.e., by multiplying Equation 1 with 10^3^:

1 $${\rm{\delta }}{\left( {{\rm{x}}/{{28}_{{\rm{Si}}}}} \right)_{{\rm{NBS}}\,28}} = \left[ {\frac{{{{\left( {\frac{{{\,}^{\rm{x}}{\rm{Si}}}}{{{\,}^{28}{\rm{Si}}}}} \right)}_{{\rm{sample}}}}}}{{{{\left( {\frac{{{\,}^{\rm{x}}{\rm{Si}}}}{{{\,}^{28}{\rm{Si}}}}} \right)}_{{\rm{NBS}}\,28}}}} - 1} \right],$$

where x denotes 29 or 30. We abbreviate δ(^x/28^Si) _NBS 28_*10^3^ as δ^x^Si and report average δ-values obtained from between 4 and 7 replicate measurements of the same analyte solution and their 95% confidence interval (=*t*∙SD/√*n*), which indicates instrument repeatability. Quality control standards were regularly measured and yielded results in agreement with published data (Supplementary Data 8). Results from solution Si stable isotope analyses are compiled in Supplementary Data 4.

### Organic carbon concentration and isotope analysis

The TOC concentration and δ^13^C_org_ were determined on decarbonated samples using an elemental analyzer NA1500 at GFZ Potsdam. Decarbonation was achieved by adding adequate volumes of 20% HCl to the sample powder in silver capsules and heating to dryness at 75 °C. Calibration was performed using an in-house urea standard and a certified standard (IAEA CH-7). The analytical precision for TOC concentrations was < 2%. The accuracy of carbon isotope analyses is estimated by repeat measurements of an internal soil reference sample (HEKATECH Boden3) that yielded accurate results, reproducible within 0.3‰ (1 SD).

### Major- and trace element analysis

Major- and trace elements were determined using X-ray fluorescence analysis (XRF) and inductively coupled plasma mass spectrometry (ICP-MS). XRF analyses were carried out on powdered and fused samples using a Philips Panalytical PW2400 at the Institute of Applied Geosciences at the Technical University Berlin. The accuracy of XRF analyses was better than 5% RSD for most elements estimated using reference materials JR-1 and JR-2 (rhyolite). The uncertainty on trace element concentrations determined by ICP-MS (Actlabs, Canada) was estimated be < 10% based on analyses of reference materials DNC-1 (dolerite), W-2a (diabase), and BIR-1a (basalt). The uncertainty of REE + Y analyses is typically < 10% RSD, estimated from analyses of reference materials DNC-1, W-2a, BIR-1a and NCS DC70014 8 (ore).

We calculate Ce* for Ce-anomalies (Ce_N_/Ce*_N_) according to Lawrence et al.^70^ (Equation 2). Using Nd and Pr to calculate Ce* precludes artificial anomalies that may arise from elevated La concentrations that result from the higher stability of La relative to other REE in solution. Ce-anomalies result from Ce(III) to Ce(IV) oxidation and enhanced scavenging onto reactive surfaces such as Fe-Mn-oxyhydroxides. As a result, Ce is becoming depleted in residual seawater and authigenic mineral phases precipitated thereof.

2 $${\rm{Ce}}_{\rm N}^* = {\rm{P}}{{\rm{r}}_{\rm{N}}} \cdot \left( {\frac{{{\rm{P}}{{\rm{r}}_{\rm{N}}}}}{{{\rm{N}}{{\rm{d}}_{\rm{N}}}}}} \right).$$

The subscript N denotes element concentrations normalized to post PAAS^53^.

We calculate the Ge/Si ratio of illite based on the illite stoichiometry, measured bulk rock Al_2_O_3_ concentrations and based on constant Ge/Si ratios of the pure silica end-member of 0.5 (Supplementary Fig. 2).

We report normalized element concentrations as element (El) enrichment factors (EF), defined as:

3 $${\rm{E}}{{\rm{l}}_{{\rm{EF}}}} = \frac{{E{l_{\rm N}}}}{{A{l_{\rm N}}}}.$$

To preclude artefacts from normalization, EFs were calculated only for samples with  > 3% detritus^43^. Moreover, we note that coefficients of variation are typically smaller for Al than for the redox-sensitive elements reported (Cu, Ni, U, V, Cr, P and Ba) except Mo.

### Mineralogical composition

X-ray diffraction analyses (XRD) were carried out at the Institute of Applied Geosciences at the Technical University Berlin and GFZ Potsdam. Powdered samples were analyzed using an Iso-Debyeflex Philips PW 1050 diffractometer or a Panalytical Empyrean, respectively, both equipped with Cu-sources. XRD patterns were measured between 5–80° 2-theta and 2.5–80° 2-theta, respectively. Mineral phase composition and quantification were evaluated with the software X’Pert HighScore, and Autoquan. Uncertainties are estimated to < 10 wt% for major mineral constituents.

### Siliceous sponge spicule abundance by mass balance

To quantify the abundance of Si derived from siliceous sponges we assume that the sampled cherts consist of four major Si sources. These Si sources are inorganic silica precipitated from seawater, Si in clay, Si in detrital quartz, and Si derived from sponge spicules. We formulate a Si stable isotope mass balance equation to calculate the abundance of sponge spicules in each sample:

4 $$\begin{array}{ccccc}\\ {{\rm{\delta }}^{30}}{\rm{S}}{{\rm{i}}_{{\rm{chert}}}} = & \left( {{\rm{f}}{{({\rm{Si}})}_{{\rm{detr}}}} + {\rm{f}}{{({\rm{Si}})}_{{\rm{auth}}}}} \right) \cdot {{\rm{\delta }}^{30}}{\rm{S}}{{\rm{i}}_{{\rm{clay}}}}\hfill\\ \\ & + {\rm{f}}{({\rm{Si}})_{{\rm{qtz}}}} \cdot {{\rm{\delta }}^{30}}{\rm{S}}{{\rm{i}}_{{\rm{qtz}}}} + {\rm{f}}{({\rm{Si}})_{{\rm{inorg}}}} \cdot {{\rm{\delta }}^{30}}{\rm{S}}{{\rm{i}}_{{\rm{inorg}}}}\\ \\ & + {\rm{f}}{({\rm{Si}})_{{\rm{sponge}}}} \cdot {{\rm{\delta }}^{30}}{\rm{S}}{{\rm{i}}_{{\rm{sponge}}}},\hfill\\ \end{array}$$

where ‘detr’ and ‘auth’ denote detrital and authigenic clay, ‘qtz’ detrital quartz, ‘inorg’ seawater Si, and ‘sponge’ Si derived from siliceous sponge spicules. We derive the fractional contribution of clay (illite) from Al concentrations, validated by quantitative XRD analyses on selected samples (see Supplementary Data 7) and assume proportional detrital quartz abundances (see Supplementary Note 2). We assign isotope compositions and uncertainties to all compartments, and solve the equation for f(Si)_sponge_. We define the fraction of sponge-derived Si relative to the sum of Si derived from non-detrital components as f_sponge_. We assign the isotope composition of clay (detrital and authigenic) and detrital quartz the values of −0.8 ± 0.3‰ δ^30^Si and −0.3 ± 0.3‰ δ^30^Si, respectively. Seawater δ^30^Si is assumed to be 1.1 ± 0.5‰, the average of inorganic seawater silica precipitates from the Precambrian (avg.: 0.83‰ δ^30^Si; Ramseyer et al.^21^) and modern river water (avg.: 1.28‰ δ^30^Si; Frings et al.^39^). The sponge δ^30^Si is estimated to be −4.9‰ based on constant Si stable isotope fractionation by modern sponges at high Si concentrations modelled to be ε^30^Si = −6.02‰^22^. We assume that also the isotope composition of the inorganic seawater precipitate remained constant during the Precambrian–Cambrian transition within certain ranges. To account for possible species-dependent variations, we assume an uncertainty of 1‰ on the sponge δ^30^Si and 0.5‰ on seawater δ^30^Si. Resulting uncertainties on f_sponge_ values were estimated using a Monte Carlo (MC) error propagation technique. For details about the mass balance approach see the Supplementary Note 2.

### Data availability

The data that support the findings of this study are available within the paper and its Supplementary Information files.

## Electronic supplementary material

Supplementary Information

Supplementary Data 1

Supplementary Data 2

Supplementary Data 3

Supplementary Data 4

Supplementary Data 5

Supplementary Data 6

Supplementary Data 7

Supplementary Data 8
