# Supplementary material for: Late Neoproterozoic seawater oxygenation by siliceous sponges
Source: Nat Commun. 2017 Sep 20;8:621. doi: 10.1038/s41467-017-00586-5 (PMC5606986; doi:10.1038/s41467-017-00586-5)
Supplement: Supplementary file 1 — Supplementary Information [file 41467_2017_586_MOESM1_ESM.pdf]

Description of Supplementary Files

File Name: Supplementary Information
Description: Supplementary Figures, Supplementary Notes and Supplementary References

File Name: Supplementary Data 1
Description: Total carbon (TC) concentrations, total organic carbon (TOC) concentrations and carbon isotope data of chert and siliceous shales of Lijiatuo section as analyzed by an elemental analyzer (NA1500). Uncertainties on C concentrations are < 2 % and approx. 0.3 ‰. on $\delta^{13}C_{org}$.

File Name: Supplementary Data 2
Description: Major element concentration (XRF) and mineralogical composition (XRD) of bulk chert and siliceous shales from Lijiatuo section. *XRD analyses: quartz is present as main component in all samples. Ba= barite, Kfs= K-feldspar, Il= illite, Py= pyrite, BaFs= Ba-feldspar, 15 Å = 15 Å clay mineral. Brackets indicate abundances < 5 wt%. The uncertainty of XRF analyses was better than 5 % RSD for most elements and < 22 % for MgO and TiO_2 estimated based on analyses of reference materials JR-1 and JR-2.

File Name: Supplementary Data 3
Description: Trace elements concentrations and Ge/Si of bulk chert and siliceous shales of Lijiatuo section as analyzed by ICP-MS (Actlabs, Canada). The uncertainty on trace element concentrations determined by ICP-MS was estimated to < 10 % based on analyses of reference materials DNC-1, W-2a, and BIR-1a.

File Name: Supplementary Data 4
Description: Silicon isotope data of bulk chert and siliceous shales of Lijiatuo section as analyzed by MC-ICP-MS. Average δ-values of n replicate measurements and their 95 % confidence intervals (= t·SD/√n) are reported (intermediate precision of the mass spectrometric method, instrument repeatability). The uncertainty of the solution MC-ICP-MS method (external long-term repeatability) is estimated to ± 0.07 ‰ (2 SD) for $\delta^{29}Si$ and ± 0.10 ‰ (2 SD) for $\delta^{30}Si$.

File Name: Supplementary Data 5
Description: Concentration of rare earth elements in bulk chert and siliceous shales of Lijiatuo section as analyzed by ICP-MS (Actlabs, Canada). Ce_N/Ce^*_N, Eu_N/Eu^*_N, and Pr_N/Yb_N are calculated according to Lawrence et al.1 from PAAS-normalized 2 element concentrations. The uncertainty of REE+Y analyses is typically < 10 %, as estimated from analyses of reference materials DNC-1, W-2a, BIR-1a and NCS DC70014.

File Name: Supplementary Data 6
Description: Estimated uncertainty of fsponge based on a Monte Carlo error propagation technique. The sponge $\delta^{30}Si$ end-member is assumed to be -3.9 ‰, according to seawater at 1.1 ‰ $\delta^{30}Si$ and a $\Delta^{30}Si$ (sponge-seawater)= -5 ‰. The 25 % and 75 % percentiles (yielding a 50 % confidence interval) of the output distribution from 1,000,000 runs are reported.

File Name: Supplementary Data 7
Description: Illite mass fractions of some samples as determined by quantitative XRD measurements and calculated from bulk rock Al/Si based on assumptions discussed in Supplementary Note 2. The uncertainty on calculated Al/Si is estimated to 7 %; on quantitative XRD analyses to < 10 %.

File Name: Supplementary Data 8
Description: Silicon isotope data of solution MC-ICP-MS measurements of reference materials obtained during this study. Individual measurements from different analytical sessions are listed. Average δ-values and their 95 % confidence interval (= t·SD/√n), are calculated (intermediate precision of the mass spectrometric method, instrument repeatability). The uncertainty of the solution MC-ICP-MS method (external long-term repeatability) is estimated to ± 0.07 ‰ (2 SD) for δ^{29}Si and ± 0.10 ‰ (2 SD) for δ^{30}Si. Published values of reference materials are within uncertainty identical to values measured during this study. BigBatch: δ^{29}Si= -5.35 ± 0.15 ‰ (1 SD, average of 11 published average values 1), δ^{30}Si= -10.48 ± 0.27 ‰ (1 SD, average of 11 published average values1); Diatomite: δ^{29}Si= 0.64 ± 0.02 ‰ (1SD, average of 9 published average values reported on GeoREM 2), δ^{30}Si= 1.24 ± 0.06 ‰ (1 SD, average of 15 published average values reported on GeoREM 2); IRMM-17: δ^{29}Si= -0.69 ± 0.04 ‰ (1 SD, average of 3 published average values reported on GeoREM 2), δ^{30}Si= -1.33 ± 0.08 ‰ (1 SD, average of three published average values reported on GeoREM 2 ; BHVO-2: δ^{29}Si= -0.14 ± 0.04 ‰ (1 SD, average of 12 published values 1), δ^{30}Si= -0.28 ± 0.05 ‰ (1 SD, average of 18 published average values 1).

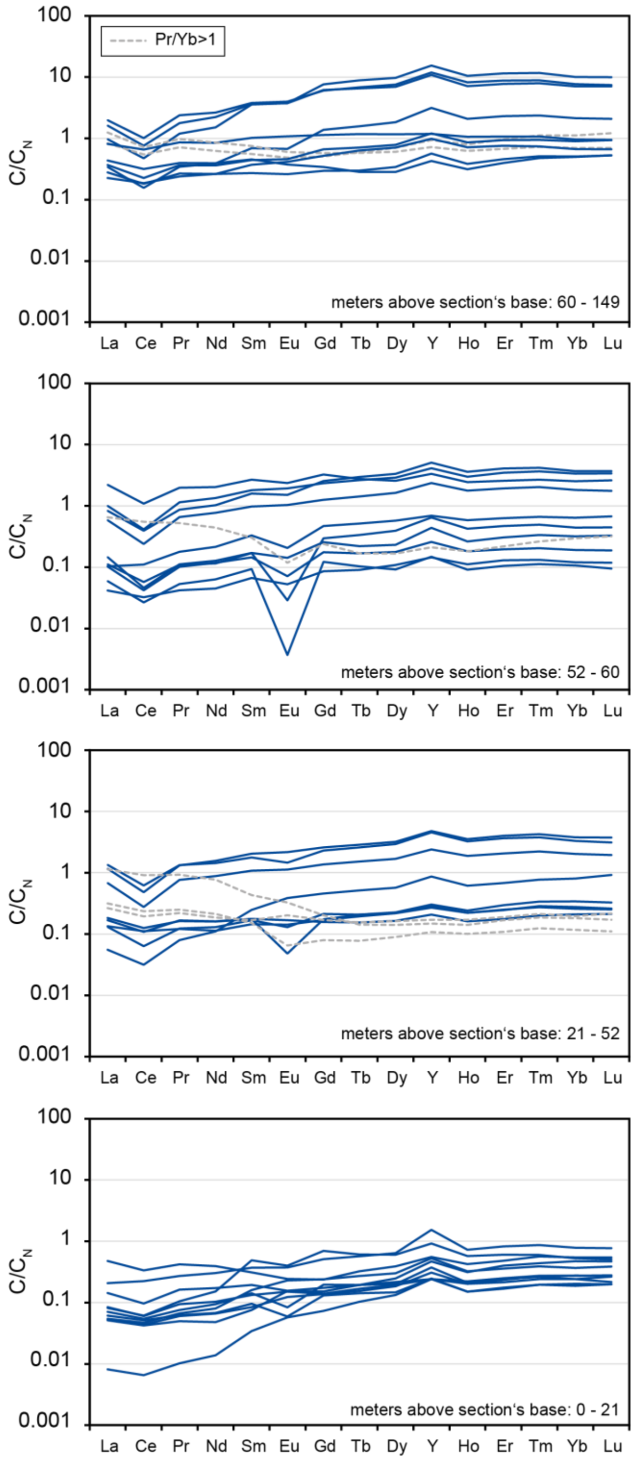

Supplementary Figure 1: Rare earth element + Y (REE+Y) patterns of bulk cherts and siliceous shales of the 'Lijiatuo' section sorted according to the height above the base of the section. The REE+Y are present in non-detrital phases (Supplementary Note 4). Samples with Pr_N/Yb_N > 1 (stippled lines) indicate REE+Y patterns different from that of seawater. Negative Eu anomalies in some samples result from overcorrection of Ba interferences of Ba-rich samples.

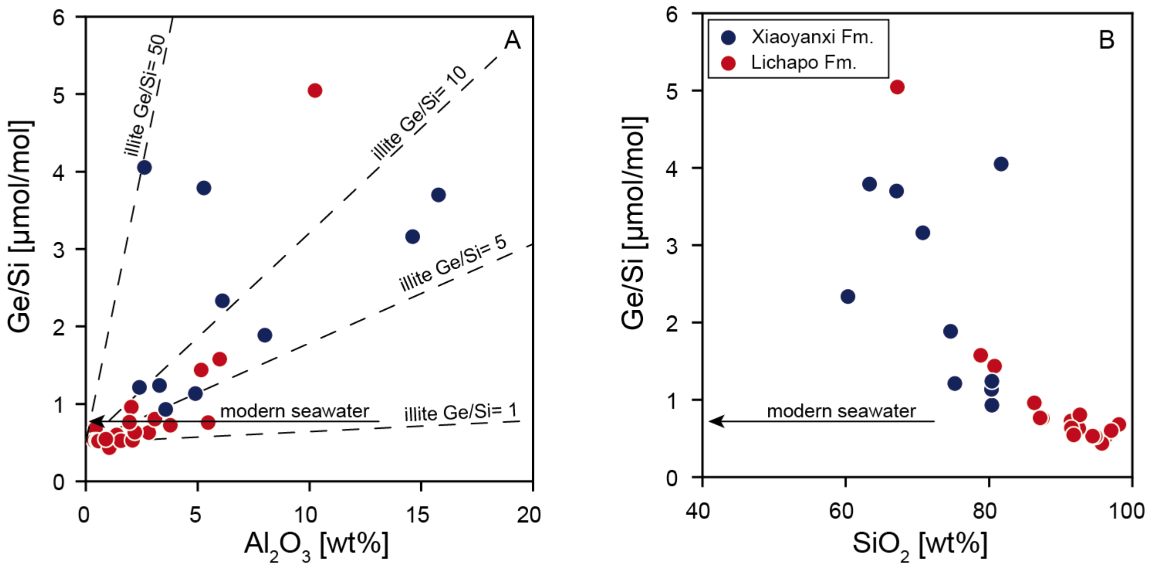

Supplementary Figure 2: Ge/Si vs. Al_2O_3 and SiO_2. Ge/Si ratios increase with increasing clay mineral content, where the illite endmember Ge/Si ratios are between approx. 5 and 50 µmol mol^{-1} (A) (cf. main text Figure 2E). Ge/Si ratios of silica end-member are similar to modern seawater (B).

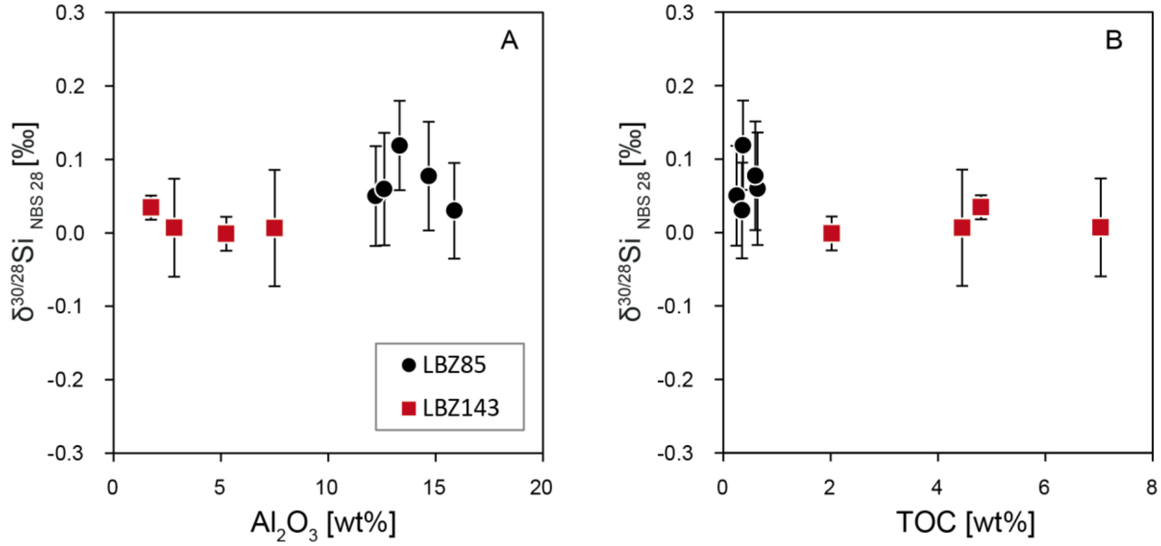

Supplementary Figure 3: Bulk chert $\delta^{30/28}Si_{NBS\ 28}$ of chert samples from two continuous chert layers (LBZ85, LBZ143). (A) $\delta^{30}Si$ vs. Al_2O_3 concentration and (B) $\delta^{30}Si$ vs. TOC concentration of samples collected laterally, approximately 0.7 to 1 m apart, along distinct chert beds at 'Longbizui' section, South China. Al_2O_3 and TOC display a wide range, but $\delta^{30}Si$ remains constant, indicating that the silicon isotope fractionation factor during precipitation was not affected by variations of clay mineral and organic carbon concentrations. Error bars represent the 95 % confidence interval of mass spectrometric replicate analyses (n= 4).

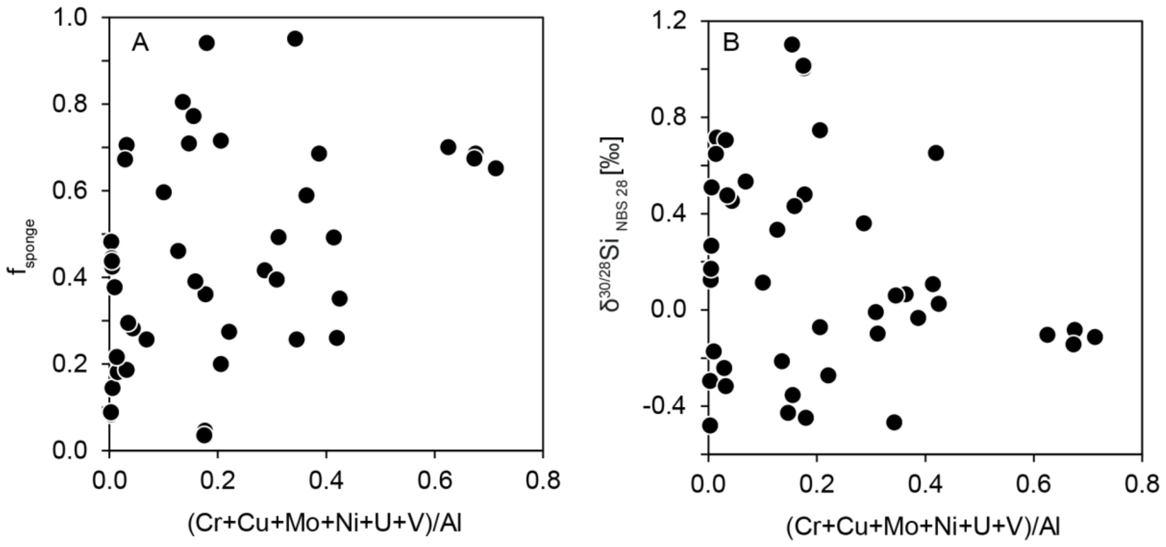

Supplementary Figure 4: Al-normalized trace element concentration vs. sponge abundance (f_{sponge}) and $\delta^{30/28}Si_{NBS\ 28}$ of bulk chert from Lijiatuo section. Neither f_{sponge} (A) nor $\delta^{30}Si$ (B) shows a clear variation with trace metal enrichment, suggesting that Si stable isotope fractionation during possible adsorption onto Fe-Mn-oxyhydroxides (that might cause trace element enrichment in sediment) does not exert the dominant control over $\delta^{30}Si$.

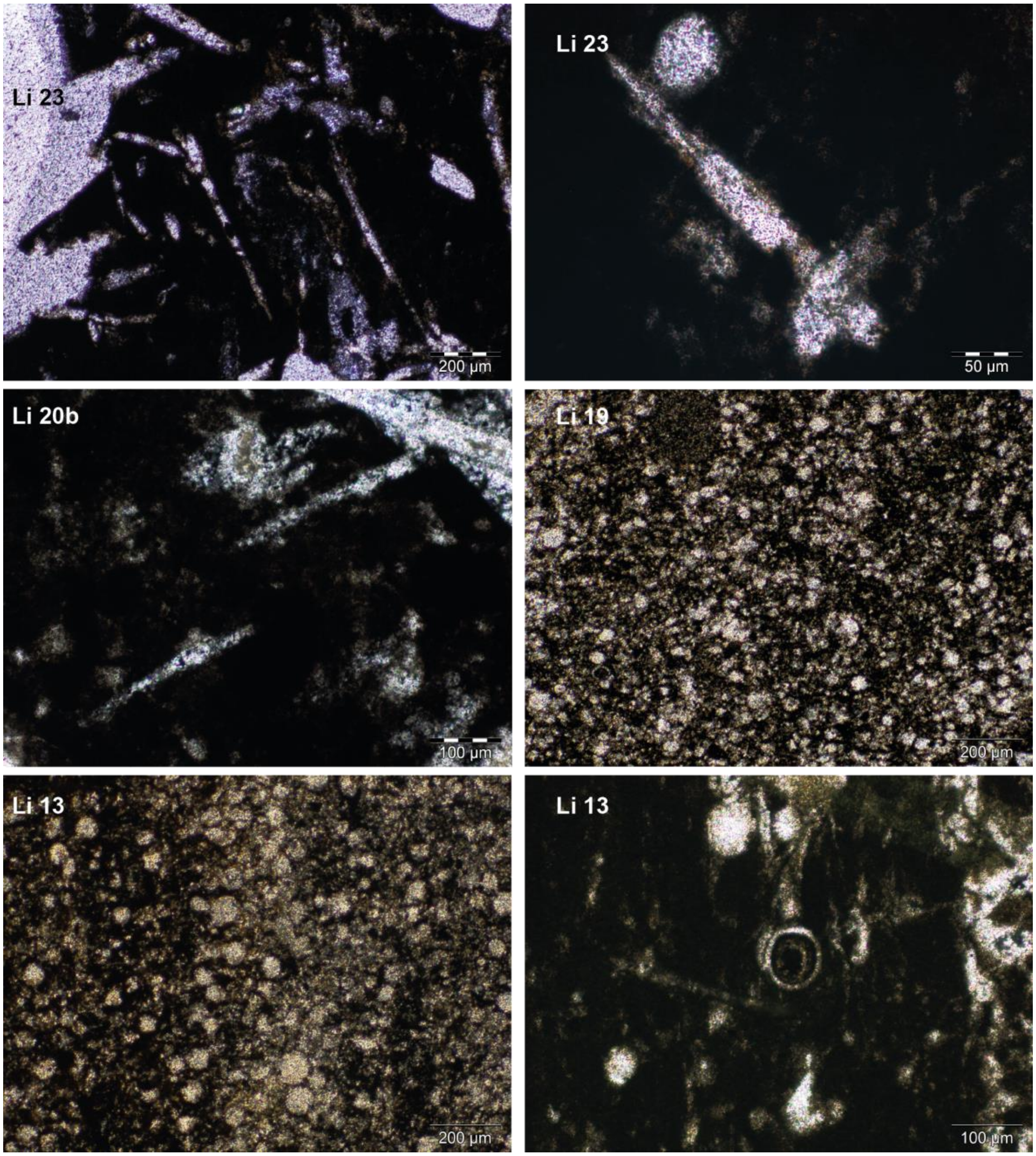

Supplementary Figure 5: Thin section photographs (transmitted light microscopy) of siliceous sponge spicules and spherical objects (putative radiolarians).

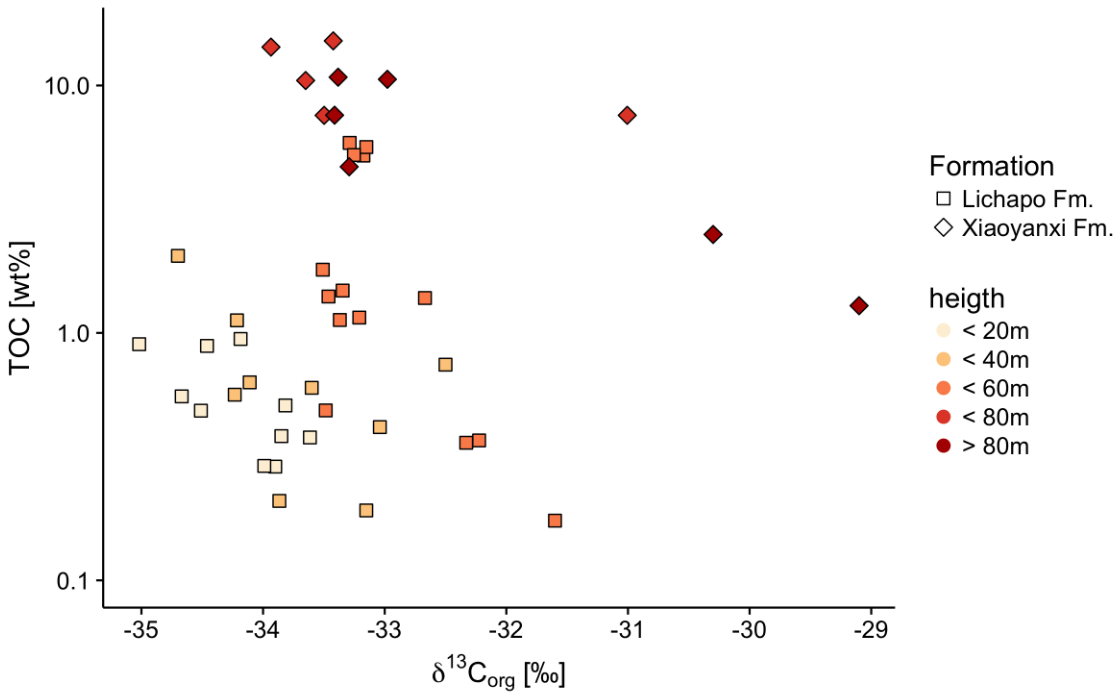

Supplementary Figure 6: Variation of TOC vs $\delta^{13/12}C_{org\ V\text{-}PDB}$ for different levels of the stratigraphic section. Within each given part of the section, TOC and $\delta^{13}C$ are anti-correlated. Across the section, TOC and $\delta^{13}C$ increase.

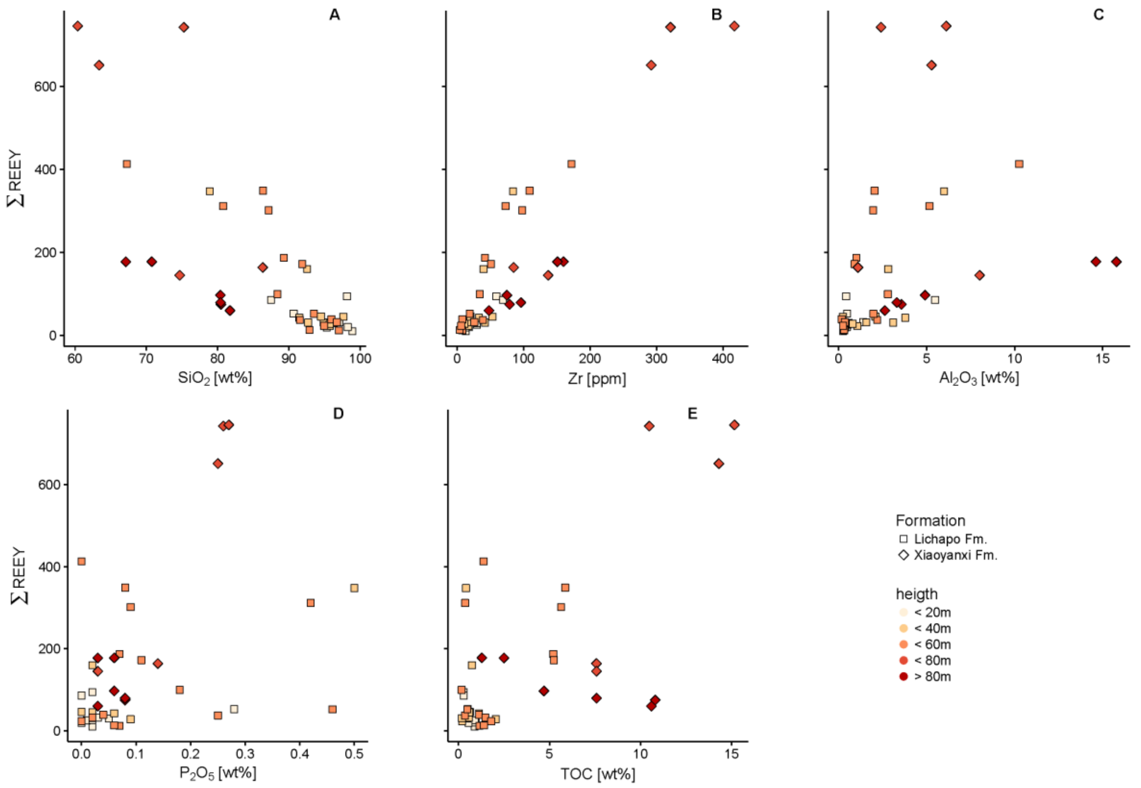

Supplementary Figure 7: Total rare earth element and yttrium (ΣREE+Y) concentrations vs. SiO_2 (A), Zr (B), Al_2O_3 (C) P_2O_5 (D) TOC (E). Decreasing ΣREE+Y concentrations with increasing SiO_2 concentrations show that silica contains very low concentrations of ΣREE+Y (A). ΣREE+Y are well correlated with Zr and Al_2O_3 (B, C). This correlation with elements derived from clastic components (zircon, clay) suggests that ΣREE+Y scavenging occurs at the seawater-sediment interface rather than in the water column (see main text for explanations). ΣREE+Y are poorly correlated with P_2O_5 and TOC concentrations (D, E).

Supplementary Note 1: Constant silicon isotope fractionation during silica precipitation

The bulk sediment $\delta^{30}Si$ could potentially be dependent on the composition of the sediment and thus sedimentary facies, because clay and organic matter can affect rates of silica precipitation [1,2] and thus Si isotope fractionation [2]. To rule out that the changing sedimentary facies towards more clay- and organic matter rich deposits affected the $\delta^{30}Si$ trend upsection, we have compared the within-layer variability of Al_2O_3 and TOC with $\delta^{30}Si$. We have collected nine chert samples from two continuous stratigraphic layers at 'Longbizui' section, Hunan Province South China (28°30'0.00"N, 109°50'24.00"E). Individual samples were collected 0.7 to 1 m apart from one another, with a total lateral range between 2.7 to 4.1 m. The bulk chert $\delta^{30}Si$ of all samples within the chert layers is analytically indistinguishable despite a large range of Al_2O_3 and TOC (Supplementary Fig. 3). Therefore, we conclude that the fractionation factor during silica precipitation was not affected by variable clay and organic matter concentrations. Given the REE and Ge/Si evidence against a hydrothermal component (see main text), we interpret the changes in $\delta^{30}Si$ of silica in the 'Lijiatuo' section to indicate changes in the relative abundance of inorganically precipitated silica and siliceous sponge spicules (main text, Figure 2).

Si isotope fractionation during adsorption onto Fe-Mn oxyhydroxide could potentially also result in ^{28}Si enrichment in sediment [3]. However, this process cannot exclusively explain the bulk chert $\delta^{30}Si$ trend, because $\delta^{30}Si$ decreases in the lowermost part of the section (i.e. the interpreted initial increase in siliceous sponge abundance) where trace element enrichment is not observed. If Fe-Mn-oxyhydroxide shuttling, i.e. the particulate transfer of elements to depth, would exert the dominant control on $\delta^{30}Si$, trace metals would be enriched here.

The lack of correlation between normalized trace element concentration and $\delta^{30}Si$ or f_{sponge} (Supplementary Fig. 4) supports this inference. Moreover, petrographical evidence supports a siliceous sponge Si source.

Supplementary Note 2: Geochemical mass balance

The sampled chert consists of silica inorganically precipitated from seawater, detrital minerals (clays: “detr” and quartz: “qtz”), authigenic illite (“auth”), and silica from sponge spicules. The chemical and Si isotope composition of bulk samples is a mixture between five end members (see below), the relative fractions of which are expressed here in Si mass fractions f. In order to calculate the contribution of sponge material in each sample, the following mass balance equations can be written.

$$\begin{aligned}\delta^{30}\mathrm{Si}_{\mathrm{chert}} = {} & (\mathrm{f(Si)}_{\mathrm{detr}} + \mathrm{f(Si)}_{\mathrm{auth}}) \cdot \delta^{30}\mathrm{Si}_{\mathrm{clay}} \\ & + \mathrm{f(Si)}_{\mathrm{qtz}} \cdot \delta^{30}\mathrm{Si}_{\mathrm{qtz}} \\ & + \mathrm{f(Si)}_{\mathrm{inorg}} \cdot \delta^{30}\mathrm{Si}_{\mathrm{inorg}} \\ & + \mathrm{f(Si)}_{\mathrm{sponge}} \cdot \delta^{30}\mathrm{Si}_{\mathrm{sponge}}\end{aligned} \qquad \text{[Equation 1]}$$

The dominant detrital mineral of the siliceous shales and chert is illite (Supplementary Data 2) and thus for simplification we assume that Al is exclusively derived from illite. This assumption is verified by exemplary quantitative XRD analyses, showing that this approximation yields calculated amounts of illite that are within uncertainty identical to measured amounts (Supplementary Data 7). Furthermore, we assume that detrital illite (detr) and authigenic illite (auth) are present.

$$\left(\frac{\mathrm{Al}}{\mathrm{Si}}\right)_{\mathrm{chert}} = \mathrm{f(Si)}_{\mathrm{detr}} \cdot \left(\frac{\mathrm{Al}}{\mathrm{Si}}\right)_{\mathrm{detr}} + \mathrm{f(Si)}_{\mathrm{auth}} \cdot \left(\frac{\mathrm{Al}}{\mathrm{Si}}\right)_{\mathrm{auth}} \qquad \text{[Equation 2]}$$

where $\left(\frac{\mathrm{Al}}{\mathrm{Si}}\right)_{\mathrm{j}}$ and $\delta^{30}\mathrm{Si}_{\mathrm{j}}$ are the molar Al/Si ratio and Si isotope ratio, respectively, of the bulk sample (j= chert) or end member (quartz: j= qtz, detrital clays: j= detr, authigenic clays: j= auth, inorganically precipitated silica: j= inorg, and sponge silica: j= sponge), and $\mathrm{f(Si)}_{\mathrm{j}}$ is the fraction of bulk Si that is present in the end member j, such that:

$$f(Si)_{detr} + f(Si)_{auth} + f(Si)_{qtz} + f(Si)_{inorg} + f(Si)_{sponge} = 1 \qquad \text{[Equation 3]}$$

The authigenic clay represents illite that formed during burial diagenesis from smectite. During the diagenetic transformation reaction of K-feldspar and smectite to illite two moles of SiO_2 are released per mole illite formed [6].

Additional constraints can be used for this mass balance. As shown in equation 4, we assume that detrital clays and authigenic clays have the same Si isotope composition (see discussion below). A fraction of the illite in the chert is likely of detrital origin rather than authigenic:

$$f(Si)_{detr} = r_1 \cdot f(Si)_{auth} \qquad \text{[Equation 4]}$$

with r_1 the ratio between the number of Si moles carried by detrital illite and that carried by authigenic illite. The rest of the detrital material is quartz:

$$f(Si)_{detr} + f(Si)_{auth} = r_2 \cdot f(Si)_{qtz} \qquad \text{[Equation 5]}$$

with r_2 the ratio between the number of moles Si carried by illite over that carried by quartz.

Our mass balance problem is thus a system of 5 equations (eqs. 1 to 5) and 5 unknowns (Si fractions f(Si)) that can be solved analytically provided that constraints are available for the remaining parameters (Si isotope composition and Al/Si ratios of chert samples and of end members, as well as for r_1 and r_2). Values for $\left(\frac{Al}{Si}\right)_{chert}$ and $\delta^{30}Si_{chert}$ were taken from measurements and associated analytical uncertainties. We assume the Al/Si of detrital illite to be $\left(\frac{Al}{Si}\right)_{detr} = 0.791 \pm 0.158$ considering a typical stoichiometry of $K_{0.65}Al_{2.65}Si_{3.35}O_{10}(OH)_2$ [7] and assume a 20 % uncertainty on this element ratio. For authigenic illite we use $\left(\frac{Al}{Si}\right)_{auth} = 0.495 \pm 0.099$ (20 %), accounting for the two moles SiO_2 that are

generated during diagenetic smectite- to illite conversion [6]. As the SiO_2 released in this reaction and in the authigenic illite is twice as high as that in detrital illite, $r_1 = 0.5 \pm 0.5$. Fine-grained clastic sediments also contain a fraction of detrital quartz, which is difficult to quantify. We assume that the clay/detrital quartz ratio is 1.7 [8]. In terms of the ratio of moles Si in illite over moles Si in quartz, this yields $r_2 = 1.0 \pm 1$. For the sponge ($\delta^{30}\mathrm{Si}_{\mathrm{sponge}}$) and inorganic silica ($\delta^{30}\mathrm{Si}_{\mathrm{inorg}}$) end-members we use -3.9 ± 0.5 δ^{30}Si and 1.1 ± 0.5 ‰ δ^{30}Si; for clay $\delta^{30}\mathrm{Si}_{\mathrm{clay}}$ and detrital quartz $\delta^{30}\mathrm{Si}_{\mathrm{qtz}}$ we use -0.80 ± 0.3 ‰ δ^{30}Si and -0.1 ± 0.3 ‰ δ^{30}Si, respectively. These values are justified in sections below. We report the detritus-free-normalized fraction of Si derived from sponges, f_{sponge}, i.e., $\mathrm{f(Si)}_{\mathrm{sponge}} / (\mathrm{f(Si)}_{\mathrm{inorg}} + \mathrm{f(Si)}_{\mathrm{sponge}})$ (see main text).

Uncertainties on f_{sponge} values were estimated using a Monte Carlo (MC) error propagation technique. We consider that two types of input parameters should be distinguished for the purpose of the present mass balance: (a) composition of the end members (Si isotope composition and Al/Si ratios) and r_1 and r_2 ratios, which might not vary greatly from a sample to another if environmental conditions do not shift significantly over the time covered by the stratigraphic record; (b) composition of samples (Si isotope composition and Al/Si ratios) which by definition vary from a sample to another, even for given environmental conditions. Therefore, we follow a "nested" MC approach where during each of 1,000 runs, a set of values was picked randomly for the parameters (a), assuming that each parameter was normally distributed (with mean and standard deviation as indicated in the previous paragraph). For each of these runs, 1,000 runs were made for each sample of the stratigraphic record, this time picking randomly values for the parameters (b), assuming a Student's distribution and using their analytical confidence interval (Supplementary Data 6). By doing so, we obtain 1,000,000 runs over which the values of $\left(\frac{\mathrm{Al}}{\mathrm{Si}}\right)_{\mathrm{chert}}$ and $\delta^{30}\mathrm{Si}_{\mathrm{chert}}$ are picked up independently across the sample set (no covariance), but with a certain degree of covariance between samples regarding the end member compositions. We emphasise that uncertainty results were only marginally

different using directly 1,000,000 runs over which all variables were considered independent, but believe that our approach is in theory sounder. Similar results were also obtained with lower number of simulations (10,000 in total), providing confidence that our simulations are statistically significant. Results are reported as the median and 25th and 75th percentiles (hence yielding a 50 % confidence interval) of the output distribution over the 1,000,000 runs.

Detrital quartz and clay have comparably well-known Si isotope compositions. Because most detrital quartz has an igneous or metamorphic source, its isotope composition is relatively uniform. Clay δ^{30}Si bears a greater variability, where clay δ^{30}Si is typically negative [9–11]. The $\delta^{30}\mathrm{Si}_{\mathrm{qtz}}$ was set to -0.1 ± 0.2 ‰ δ^{30}Si, overlapping with average values determined for loess (comprising both quartz and feldspar) of -0.22 ‰ δ^{30}Si [12]; for clay we assume -0.8 ‰ δ^{30}Si, according to a kaolinite-rich mudstone (Li173; Supplementary Data 4) and the clay Si isotope composition extrapolated from the shale sample with the highest Al-content (Li 31; Supplementary Data 2, 4).

Sponge silica is isotopically distinct from seawater through preferential uptake of light silicon. Modern sponges fractionate Si isotopes with up to $\alpha_{(\text{sponge-seawater})}$= 0.995 [13,14] where Si isotope fractionation by modern sponges reveals a dependence on seawater silicon concentrations between ca. 1.7 ppm (ca. 60 µmol/L) [14] and ca. 4.2 ppm (150 µmol/L) [13]. Isotope fractionation is believed to asymptotically attain a constant value of $\alpha_{(\text{sponge-seawater})}$= 0.994 at very high Si concentrations [14]. Therefore, we predict that small variations in Si concentration in Late Neoproterozoic seawater would not affect the magnitude of Si isotope fractionation by sponges. We therefore assume a constant δ^{30}Si endmember value for sponge spicules in the ‘Lijiatuo’ section. Because $\alpha_{(\text{sponge-seawater})}$ of Precambrian sponges is unknown, we report $\mathrm{f(Si)}_{\mathrm{sponge}}$ for sponge end-member Si isotope compositions of -4.9, -2.9 ‰ and -0.9 ‰ δ^{30}Si (main text Figure 2B), corresponding to fractionation factors $\alpha_{(\text{sponge-seawater})}$ of 0.994, 0.996 and 0.998. While the absolute value of $\mathrm{f(Si)}_{\mathrm{sponge}}$ crucially depends on the unknown fractionation factor between ancient sponges and seawater, temporal trends in

the normalized f_{sponge} value are insensitive to the choice of the end member isotope composition. Additionally, we argue that changes in the seawater Si isotope composition induced by removing sponge Si itself are unlikely. Indeed, the Si inventory of the ancient ocean was so large that a comparatively small Si output flux by sponges was not able to significantly shift the seawater silicon isotope composition (we note that such a mass balance effect cannot be induced by inorganic silica precipitation as its $\alpha = 1$, see above). However, even if there were a shift in seawater $\delta^{30}Si$ induced by sponge spicule formation, the resulting increase in seawater $\delta^{30}Si$ would actually result in underestimated sponge abundances. Such underestimate would be most pronounced in the upper layers where f_{sponge} is highest, meaning we potentially underestimate their contribution in our current analysis. Such underestimate would leave the interpretation unchanged.

Supplementary Note 3: The TOC-$\delta^{13}C_{org}$ relation

In any given part of the section, samples with relatively lower TOC concentrations are higher in $\delta^{13}C_{org}$ relative to TOC-rich samples (Supplementary Fig. 6). There are two possible causes for this feature that are primary and/or diagenetic in nature. Sponges selectively take up small-sized organic carbon pools (fine particulate and dissolved organic carbon; POC and DOC): particle retention efficiencies are highest in the size range between 0.1 and 10 µm [15]. Therefore, if organic carbon has a size-dependent carbon isotope composition sediments would become enriched in ^{12}C relative to bulk organic carbon by filter feeding through sponges. Alternatively, or additionally, a shift towards high $\delta^{13}C_{org}$ (by 2 to 3 ‰) and low TOC can result from thermal alteration due to the extraction of isotopically light hydrocarbons [16]. This effect increases the $\delta^{13}C$ of residual organic carbon, and would be pronounced in samples with low organic carbon concentrations. Overall, the isotopic difference between samples with low and high organic carbon concentrations is ambiguous with respect to discerning primary from secondary controls on $\delta^{13}C_{org}$. Regardless, the trend towards higher

$\delta^{13}C_{org}$ recorded in TOC-rich samples on the outcrop scale (main text Figure 1B) should be unaffected by potential diagenetic effects due to overall low post-depositional organic carbon losses - as suggested by the TOC-Ni and TOC-Cu correlations (main text figure 3).

Supplementary Note 4: Rare earth element geochemistry

Typical seawater REE patterns in bulk sediment (Supplementary Fig. 1) indicate that REEs were scavenged from seawater. REE uptake at the water-sediment interface, and thus Ce_N/Ce^*_N as recorder of the bottom water redox state, is suggested by the well-correlated concentrations of REE+Y with Zr and Al (Supplementary Fig. 7). The sedimentation rate dependence of Zr and REE+Y accumulation shows that REE+Y scavenging cannot predominantly occur in the water column. With decreasing burial rates, i.e. increased sediment exposure time, REE+Y are increasingly scavenged onto particles residing on the seafloor [4,5]. At the same time when the gross sedimentation flux (controlled by non-detrital components such as authigenic silica, biogenic silica and organic matter) decreases, detrital components (represented by Zr present in zircon) are increasingly accumulating in sediments.

Supplementary References

1. Siever, R. The silica cycle in the Precambrian. Geochim. Cosmochim. Acta **56,** 3265–3272 (1992).
2. Oelze, M., von Blanckenburg, F., Hoellen, D., Dietzel, M. & Bouchez, J. Si stable isotope fractionation during adsorption and the competition between kinetic and equilibrium isotope fractionation: implications for weathering systems. Chem. Geol. **380,** 161–171 (2014).
3. Delstanche, S. et al. Silicon isotopic fractionation during adsorption of aqueous monosilicic acid onto iron oxide. Geochim. Cosmochim. Acta **73,** 923–934 (2009).
4. Murray, R. W., Buchholtz ten Brink, M. R., Gerlach, D. C., Price Russ III, G. & Jones, D. L. Rare earth, major, and trace elements in chert from the Franciscan Complex and Monterey Group, California : Assessing REE sources to fine-grained marine sediments. Geochim. Cosmochim. Acta **55,** 1875–1895 (1991).
5. German, C. R., Klinkhammer, G. P., Edmond, J. M., Mitra, A. & Elderfield, H. Hydrothermal scavening of rare-earth elements in the ocean. Nature **345,** 516–518 (1990).
6. Abercrombie, H. J., Hutcheon, I. E., Bloch, J. D. & de Caritat, P. Silica activity and the smectite-illite reaction. Geology (1994).
7. www.mindat.org/min-2011.html. (2015). Available at: http://www.mindat.org/. (Accessed: 25th March 2015)
8. Shaw, D. B. & Weaver, C. E. The mineralogical composition of shales. J. Sediment. Petrol. **35,** 213–222 (1965).
9. Opfergelt, S. et al. Silicon isotopes and the tracing of desilication in volcanic soil weathering sequences, Guadeloupe. Chem. Geol. **326–327,** 113–122 (2012).
10. Opfergelt, S. & Delmelle, P. Silicon isotopes and continental weathering processes: Assessing controls on Si transfer to the ocean. Comptes Rendus Geosci. **344,** 723–738 (2012).

11. Georg, R. B., Zhu, C., Reynolds, B. C. & Halliday, A. N. Stable silicon isotopes of groundwater, feldspars, and clay coatings in the Navajo Sandstone aquifer, Black Mesa, Arizona, USA. Geochim. Cosmochim. Acta **73,** 2229–2241 (2009).
12. Savage, P. S., Bastian Georg, R., Williams, H. M. & Halliday, A. N. The silicon isotope composition of the upper continental crust. Geochim. Cosmochim. Acta **109,** 384–399 (2013).
13. Hendry, K. R. & Robinson, L. F. The relationship between silicon isotope fractionation in sponges and silicic acid concentration: Modern and core-top studies of biogenic opal. Geochim. Cosmochim. Acta **81,** 1–12 (2012).
14. Wille, M. et al. Silicon isotopic fractionation in marine sponges: A new model for understanding silicon isotopic variations in sponges. Earth Planet. Sci. Lett. **292,** 281–289 (2010).
15. Maldonado, M., Ribes, M. & van Duyl, F. C. Nutrient Fluxes Through Sponges. Biology, Budgets, and Ecological Implications. Advances in Marine Biology **62,** (Elsevier Ltd., 2012).
16. Burwood, R., Drozd, J., Halpern, H. I. & Sedivy, R. A. Carbon isotopic variations of kerogen pyrolyzates. Org. Geochem. **12,** 195–205 (1988).
